# The use of virtual reality for language investigation and learning

**DOI:** 10.3389/fpsyg.2014.01280

**Published:** 2014-11-06

**Authors:** Claudia Repetto

**Affiliations:** Department of Psychology, Catholic University of the Sacred HeartMilan, Italy

**Keywords:** embodied language, virtual agents, virtual reality, language learning, second language acquisition

Virtual Reality (VR) is a technological tool traditionally used in psychology for clinical purposes or self-empowerment: a huge number of papers have documented the validity of treatments provided or supported by VR. Most of these have illustrated protocols for the treatment of anxiety disorders: from simple phobias (Krijn et al., 2007), to panic disorders (Botella et al., 2007), post-traumatic stress disorder (Gerardi et al., 2008), and generalized anxiety disorder (Repetto et al., 2009, 2013b; Repetto and Riva, 2011); recently, VR has also been effectively employed for stress management in a non-clinical population (Gaggioli et al., 2014).

However, in the last decades, growing attention has been dedicated to this tool in the field of neuroscience (Bohil et al., 2011) for the study of spatial navigation, multisensory integration, social neuroscience, pain remediation, or neurorehabilitation. Macedonia et al. (2014) took a step in this direction, using one of the VR basic elements for the investigation of language learning. In particular, they built Intelligent Virtual Avatars (IVAs) with human appearance and assigned them the task to teach a second language to different trainee populations (adults and children). The authors evaluated the results of their studies and discussed two levels of outcome: memory performance, and IVAs' acceptability.

IVAs proved to be as effective as their human counterparts at teaching capabilities; learners memorized new words better when they performed an iconic gesture associated to the word than when they only watched the teacher performing the same gesture, whether the teacher was virtual or human. This finding can easily be interpreted within the framework of Embodied Cognition Theory (Barsalou, 2008): according to this theory, the cognitive system is no longer considered as a processor of symbols and abstract operations, but is deemed to be grounded in multimodal representations molded from human experience. In this view, cognitive processes, including language, are “embodied” in nature, since the perceptual and motor systems influence the way we construct concepts, make inferences and use language. Several proofs of this claim have been provided in the neuroscience literature: it has been found that the primary motor cortex is involved in language comprehension (Repetto et al., 2013a), and that the same neural structures needed to process sensory information are also active when processing words that embed that sensory information, such as color (Martin et al., 1995).

Considering these recent findings, the use of VR in the study of language processes becomes even more reasonable. In fact, VR can be considered an “embodied technology” for its effects on body perceptions (Riva, 2002): it is possible to use VR to induce controlled changes to the experience of the body; furthermore, the virtual environment can be enriched to the extent that it can become a plausible copy of the real world.

The first feature seems very important in the study of foreign language learning: if the association of a gesture to a word can enhance verbal memory (Macedonia et al., 2011), then VR offers a privileged medium in which to implement the training. In fact, it gives users the opportunity to see themselves moving in the environment while being comfortably seated in a chair. Thanks to different input devices, participants could virtually perform any action, even those typically not performable in an experimental or learning setting (e.g., kick a ball). Thus, future research could combine the use of IVAs with the possibility for the learners to see themselves performing the gesture illustrated by the IVA: for instance, if kicking a ball is the action to imitate, the user (by manipulating a joypad) could see in the VR his own leg and foot raising up and hitting a ball. Recently, a virtual environment (Riva et al., 2009) was employed for the study of language learning and comprehension; preliminary results pointed out that, in second language learning tasks, a virtual motion (a motion performed in the virtual world with a body part that is actually steel) associated to action words can enhance verbal memory if the environment is perceived as true-to-life. Logically, in native language tasks, the virtual action can promote language comprehension (papers in preparation).

Similarly, the possibility to create very detailed environments can support the study of language: if representations in the cognitive system are multimodal, then to investigate their properties one should recreate the multimodal experience that can trigger the process. Thus, in the future, navigation in high-quality virtual worlds, associated with the execution of gestures or actions, could possibly create the optimum experience to improve the processing of words, helping trainees to learn (in case of new language acquisition) or re-learn/consolidate information (just think about the rehabilitation of language abilities in aphasic patients). New research is needed to investigate the efficacy of these trainings, and the findings by Macedonia (Macedonia et al., 2014) testify that this line of research deserves further efforts.

As far as the IVAs' acceptability is concerned, Macedonia's data seem to support the idea that, in general, both adults and younger trainees accepted the avatar Billie as a language teacher. Addressing the issue of acceptability brings us to the concept of “uncanny valley” (Mori et al., 2012). According to Mori, the acceptability of a virtual character is not a linear function of its human likeness. In other words, the more a virtual agent resembles a human being, the more it is acceptable, until it reaches a certain degree of likeness: at this point, it is perceived as “uncanny,” losing credibility and appeal. Acceptability rises again when the level of likeness exceeds this critical point, making the character almost identical to the human form. It should be noticed that the uncanny valley effect represents a potential risk for the effectiveness of training: if the trainee recognizes the virtual agent as uncanny, he/she may be less willing to adhere to the agent's instructions, or to the training itself. Apparently, this was not the case in Macedonia's research. In fact, recent new findings have called the uncanny valley effect into question. For example, a recent study by Piwek (Piwek et al., 2014) highlighted the fact that the motion quality of a full-body animated avatar can influence the acceptability of the IVA: the characters classified in the deepest location of the uncanny valley in their static form, were judged later on as more acceptable when motion was added. This result seems to indicate that motion *per se* may help to bypass the uncanny valley. Nevertheless, the evaluation of the agent acceptability appears mandatory: when building an experiment that includes IVAs, especially for psychological investigations, researchers routinely should run pre-tests that take into account the threat of the uncanny valley effect.

In conclusion, Macedonia and colleagues opened new promising paths for the study of language learning, by combining neuroscientific knowledge and new frontiers of technology that can help address new questions, and by building powerful tools for the improvement of language abilities; the future direction is to refine the paradigms taking advantage of all the capabilities that VR offers.

## Conflict of interest statement

The author declares that the research was conducted in the absence of any commercial or financial relationships that could be construed as a potential conflict of interest.
